# Implementation of online video consultations in a regional health network: a management feasibility analysis from an orthopedic perspective

**DOI:** 10.1186/s12913-022-08352-0

**Published:** 2022-08-12

**Authors:** DA Back, K Estel, D Pförringer, S Tsitsilonis, J Bachner, C Willy, working group digitalization (DGOU), HP Becker

**Affiliations:** 1 Charité –Universitätsmedizin Berlin, Center for Musculoskeletal Surgery, Freie Universität Berlin and Humboldt-Universität Zu Berlin, Berlin, Germany; 2 Department for Traumatology and Orthopedics, Bundeswehr Hospital Berlin, Scharnhorststrasse 13, 10115 Berlin, Germany; 3 Clinic and Policlinic for Traumatology, Klinikum Rechts Der Isar, Technical University Munich, Munich, Germany; 4 WINGS Professional Studies – MBA Healthcare Program, Wismar University of Applied Sciences, Wismar, Germany

**Keywords:** Community health network, Case management, Telemedicine, Analysis, Patients

## Abstract

**Introduction:**

Regional health care networks with interfaces between clinics, general practitioners and patients can act faster when utilizing digital measures. This manuscript describes the establishment of an online video consultation service in a clinic and its broad health care region to exemplify challenges and solutions for potential future approaches from a management perspective.

**Method:**

The underlying pilot project was planned and implemented for follow-up monitoring and consultative presentation of orthopedic and trauma patients within the Bundeswehr Medical Service from 2018 to 2020. With predominantly positive evaluation results regarding quality and acceptance among users, this research investigated organizational and processual aspects including total quality management, strategic control and change management approaches.

**Results:**

The affected main and subprocesses of patient treatment could be streamlined by the project, as physician recommendations and arrangements could be accelerated and patient travel could be significantly reduced. A SWOT and portfolio analysis showed a high potential for improving existing patient treatment processes for health care enterprises via the use of digital technology. The involved staff should be strategically included at an early stage and continuously involved. By means of a PDCA cycle, the processes of the given project could be exemplarily illustrated with an outlook in the future.

**Discussion:**

It has proven successful to consciously use management approaches to establish telemedical integrated care structures in a health region. Recommendations for the strategic introduction of an online video consultation for regional network strengthening and care development for a patient-oriented increase in efficiency could be compiled.

**Supplementary Information:**

The online version contains supplementary material available at 10.1186/s12913-022-08352-0.

## Introduction

Digital transformation is increasingly influencing everyday life and, consequently, the health care system [1]. The overarching goal of these digital applications is to improve existing medical care offerings. In Germany, as well as in other European countries, relevant laws have been developed in recent years to ensure data security and promote digital health innovations [2–4].

Telemedicine, for example, can be used to offer the expertise of specialists across long distances [5]. However, it was only with fast internet connections and their corresponding devices that this technology could be further developed and established in health systems in recent years, at least regarding nationwide online video consultations (OVCs). The pioneers in this were, among others, aerial states such as Australia [6]. One of the advantages of this special form of digital interaction is the independence of the location from which patients can present themselves digitally to doctors. Additionally, there are no expenses for travel and, under certain circumstances, costs for lost working hours [7]. As another positive aspect, a reduction in waiting times for patients has been demonstrated [8]. At the same time, the use of OVCs has been shown to reduce costs for health care systems and insurance providers in various studies [9, 10]. Patients generally describe a high acceptance of such digital contact methods [11]. From a professional medical perspective, studies have shown that OVCs can be equivalent to traditional doctor visits, depending on the clinical situation [12]. For physicians, the advantages of offering an OVC can be a better involvement of patients in their treatment if they have a digital affinity and the corresponding motivation, as well as attracting new patients via digital offers [4]. On the other hand, doctors also perceive OVCs as more time-consuming than regular consultations, and some doctors share concerns about the security of data exchange. The sometimes necessary deviation from usual treatment paths therefore urges the development of new processes for procedures and therapy decisions in OVCs [10].

In the context of the COVID-19 pandemic, digital doctor-patient communication quickly grew in importance from 2020 onward. While publications reporting on the clinical use of OVCs also increasingly appeared in the field of orthopedics [11, 13, 14], there are hardly any descriptions of the processes for establishing such offers for a regional network of players (or stakeholders) in health regions. When looking at regional or community health networks with close cooperation between hospitals, general practitioners and patients, it can be assumed that these networks could act more quickly and efficient with telemedical tools as communication and collaboration support [15].

However, the development and introduction of new digital processes in existing healthcare structures will need health management tools to be applied. In this context, organizational management aims to fulfill the institution´s goals by handling adequately all the processes and resources available [16]. Regarding processes specifically, health care management is increasingly applying process management strategies, which have proven to make a difference in organizational performance and competitiveness to the industry at large [17]. Here, total quality management serves as system implemented by the management of an organization to achieve the satisfaction of customers or patients, respectively [18]. Tracking a new strategy as it is being implemented needs then strategic control, with detecting problems or changes in the premises and making necessary adjustments while action is taking place [19]. Finally, change management addresses the transition of individuals, teams, and organizations to a desired future state, with changes to the scope of a project being formally introduced and approved [20].

The aim of this research was to analyze the establishment of OVCs in an orthopedic clinic with affiliated referring troop physicians, forming a community health network. Focus was set on the perspective of organizational and process management with total quality management [21, 22], strategic control [23, 24] and change management approaches [25] to develop best practice examples for possible further approaches in this context.

## Methods

### The project under consideration

Within the framework of a special research project of the Bundeswehr Medical Service (No. 23K4-S-10 1921) and an innovation project of the Cyber Innovation Hub of the Bundeswehr (CIHBw, No. #44), the pilot introduction of OVCs in the Department of Trauma Surgery and Orthopedics of the Bundeswehr Hospital Berlin (subsequently referred to as “the hospital”) was planned and has been implemented and carried out since 2018. Funding for the project was provided by the CIHBw. A digital network was set up with regional medical facilities (comparable to civilian general practitioners, subsequently referred to as GPs) in the area of eastern Germany. After the evaluation of the initial research project at the end of 2020, OVCs were firmly integrated into existing patient processes at the hospital. The study evaluation took place after review by the responsible data protection authorities and the ethics committee (Ärztekammer Berlin, No. Eth-12/19).

### The underlying health care network

The primary treatment of patients in the Bundeswehr medical service is provided by troop physicians in regional medical facilities (to facilitate understanding, and due to their similar functions, troop physicians are referred to as “general practitioners/GPs” in this manuscript). In case of more extensive needs, they refer patients to Bundeswehr specialists, most of whom work in the five military Bundeswehr hospitals [26]. The clinics are not only responsible for specialist outpatient examinations but also play the role of gatekeepers for inpatient treatment or operative procedures. Due to the sometimes large geographical distances, this can cause long journeys for patients, as well as longer waiting times for appointments for GPs and patients.

The hospital, with its structures superordinate to the clinic under consideration, is structured according to the classic functional organizational form, with a separation of primary and secondary service provision areas (medicine, nursing, diagnostics) from the tertiary parts, such as the staff within technical and administrative process departments, as well as hospital control. The treatment of noninpatient orthopedic patients is provided by the clinic's outpatient department.

### Underlying legal national regulations

In Germany, various laws (e.g., the Digital Healthcare Act, DVG [27]) have been established since 2015 to allow OVCs to be carried out by providers certified by the National Association of Statutory Health Insurance Physicians. Prior to the coronavirus pandemic, the re-presentation and consultative initial presentation, but not the sole initial presentation of patients, was permitted to be performed digitally, which is why the latter option was not considered in this project.

### Project design for the implementation of online video consultations

The project team of the hospital and CIHBw involved the relevant institutions and structural elements. The referring GPs in the examined area were contacted both via email and telephone and invited to voluntarily participate in the project, as were the patients concerned. In some cases, also personal face-to-face communication for recruiting into the study took place. They were always informed about the whole project and its pilot character, as well as about the voluntary decision for participation and the anonymization of the given and gained data.

In the first step, the technical establishment was carried out in the clinic from August 2019 to gather experience with the processes. Patients who were regularly scheduled for "live" follow-up examinations in the clinic's outpatient department were able to voluntarily perform an additional OVC with their private devices (smartphones, tablets or computers) before their re-presentation. The quality of the findings was comparatively analyzed, and the participants were evaluated. Data in this first part of the project was gained by using 5-point Likert scaled and open questions on patients´ and doctors´ experiences and attitudes (between 26 to 28 items), asking also for technical and organizational challenges.

In the second step, GPs were given the opportunity to evaluate patients via OVCs in the clinic on a consultative basis instead of the usual "live" presentation. To enable this, technical equipment with tablets (Apple and Samsung), tablet holders (Computacenter AG & Co. OHG), headsets (Plantronics) and SIM cards (Deutsche Telekom AG) was provided beginning in March 2020. Doctors and patients were asked about their technical, professional and subjective assessments. The gained data in this second part of the project consisted of patients´, specialists´ and GPs´ experience and attitude (5-point Likert-scale and open questions, 19 to 27 items). Also, the average distance between GP/patient and hospital was recorded in kilometers.

Appointments could be made by GPs via a newly introduced OVC mobile hotline or directly online via the OVC provider certified according to national regulations (Deutsche Arzt AG, Düsseldorf, Germany).

To determine the relative utilization of OVCs compared to the clinic's regular consultations, a period over six months was chosen – with beginning of April 2020, after which the referring GPs were equipped with the technical equipment, until the end of September 2020.

### Organizational management analyses of the project

In the context of this research, experiences with the project using different tools of organizational management were evaluated and analyzed.

The framework conditions were initially examined by analyzing the types of processes. First, the core and subprocesses with important support processes in the hospital should be identified, where the OVC could be helpfully used. Additionally, within a process map, the key stakeholders should be set in relation to the most relevant processes along the patients´ health road (involving GP´s and the hospital). One further question of the planned digital changes was the extent of optimization of existing processes by OVCs and the associated digital possibilities for reducing redundant steps.

Based on the experience gained with the project, a SWOT analysis was carried out to show the positioning of OVCs in the observed clinic and the connected regional area, taking into account internal strengths and weaknesses of the involved partners within the health network, as well as external factors like opportunities and threats of the underlying health care network.

To analyze the potential of the OVC as an instrument for the medical care of patients in the setting under consideration, a BCG portfolio analysis was carried out. Here, the “market growth” (i.e. the potential of use of the focused method OVC) was set in relation to the “relative market share” (i.e. the actual use of the focused method).

Subsequently as part of the project’s implementation, important aspects of human resources recruitment were additionally evaluated in a change management analysis following the 8 focal points of Kotter [28]. This was meant to show how the OVC introduction was structured, taking into consideration the acceptance and inclusion of staff and patients.

Finally, the previous, existing and further planned management processes were analyzed with the 7 questions in process management by Stöger (see supplement 4) [29]. Additionally, the PDCA cycle described by Deming was used to elaborate the optimization potential of the measure over time [21, 22].

Data analysis of this project was performed by the authors by means of the above listed existing management tools and descriptive statistics.

## Results

A digital network was set up with 16 regional medical facilities for the consultative presentation of patients with an average distance of 126 km (6 km to 328 km) from the hospital in eastern Germany. The patients themselves had a mean travel distance of 141 km, depending on their place of residence, ranging up to 650 km in some cases. The evaluation of OVCs of the two study arms is part of other studies and is not shown here ([30]; Estel et al. The use of video consultations to support orthopedic patients’ treatment at the interface of a clinic and general practitioners, submitted).

For the relative utilization of OVCs compared to the clinic's regular consultations, the ratio of OVCs (*n* = 176) to physical outpatient visits (*n* = 2374) was 7.4%.

Direct contact of the specialists with the GPs and patients was also indicated as very helpful. By getting to know each other personally and jointly developing treatment approaches, GP colleagues were able to perceive their participation in the therapy process in a positive way (Estel et al. The use of video consultations to support orthopedic patients’ treatment at the interface of a clinic and general practitioners, submitted).

### Process analysis in the area of OVCs

The primary business process or core process relevant to OVCs from the portfolio of the participating hospital is outpatient care.

**Fig. 1:**
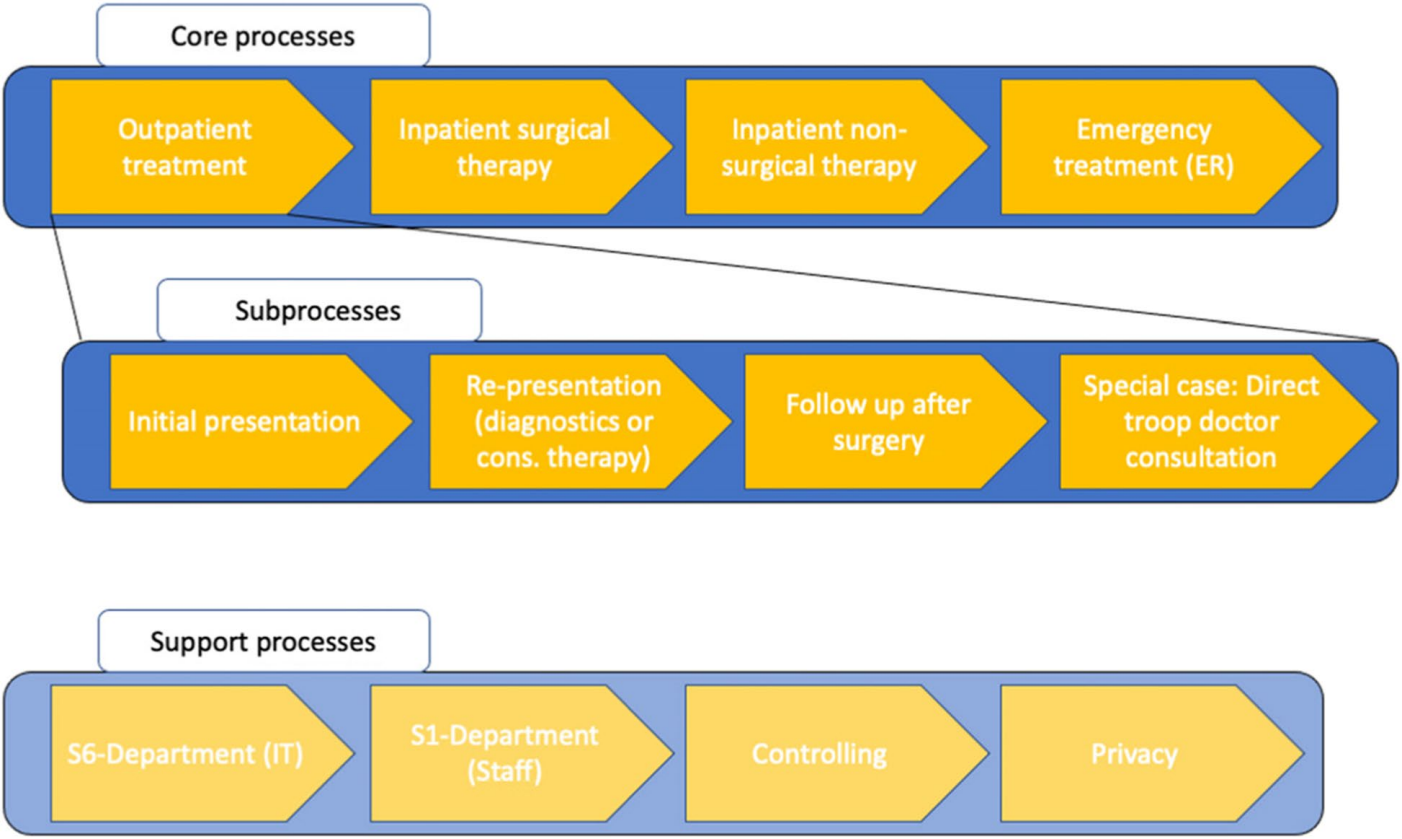
Section of a process map of core, part and support processes at Bundeswehr Hospital Berlin focusing on outpatient processes

Outpatient care is differentiated into (Fig. 1).

- 1. Initial presentation of patients with new complaints (with GPs present).
- 2. Re-presentation of outpatients after conservative therapy (mostly with GPs present).
- 3. Re-presentation of patients for follow-up after surgical interventions.
- 4. As a special case: The option of direct medical consultation without patient contact, which is, however, rarely used due to the requirement of an appointment.

**Fig. 2:**
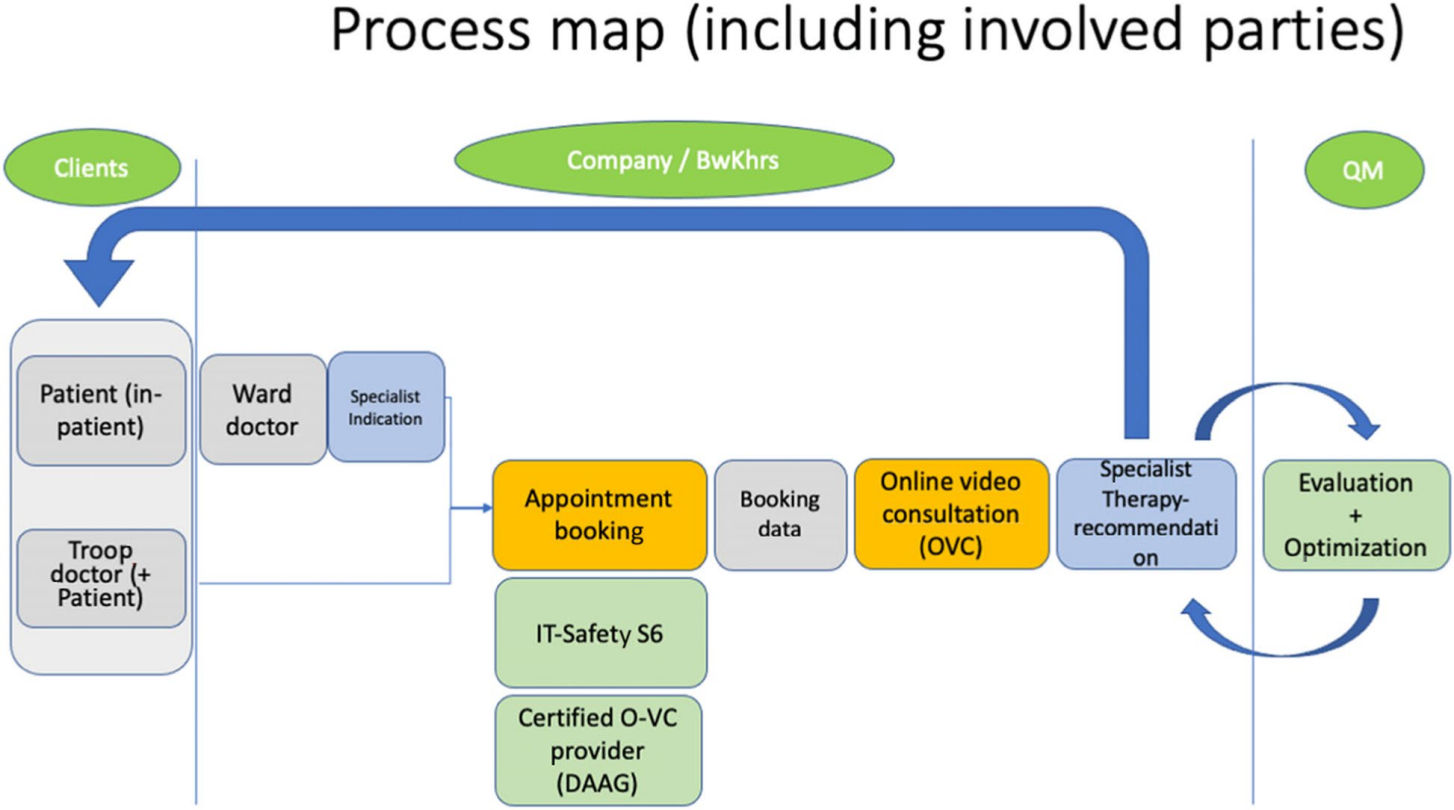
Process map including key stakeholders (Abbreviation: QM = quality management)

With the inclusion of the "clients" in the sense of patients who want a specialist's opinion on their condition or general practitioners who request a consultation and recommendation, the core, partial, support and management processes can also be depicted in a process map as a flow chart (Fig. 2).

Regarding the analysis for reduction for redundant steps in the existing processes of interdisciplinary patients´ treatment between the GPs and the hospital, especially travel savings could be identified. Supplements 1, 2, and 3 show the flowcharts of the processes of patient follow-up after inpatient treatment, GP initial presentations and re-presentations.

### SWOT analysis

A SWOT analysis of the inclusion of OVCs in the clinic under consideration and the regional area of influence is shown in Table 1.

**Table 1:** SWOT analysis for the introduction of online video consultations in orthopedics/traumatology at Bundeswehr Hospital Berlin and its regional catchment area

**SWOT**		
	**Strengths** (internal) Existing high demand due to efficiency streamlining of the existing processes for - Patients (faster decision-making, avoidance of long journeys, sometimes with complaints from the patients, necessary driver position, also often with several hours or whole-day absences from work) - GPs (faster decision-making, faster and personalized professional support) - Specialists (faster initiation of required diagnostics and, if necessary, therapy paths, concentration of capacities in the outpatient clinic for more complex cases) Motivation of the doctors involved, "unbureaucratic" funding	**Weaknesses** (internal) Thin staffing in the outpatient department at the hospital, Dependence on the acceptance of medical users Lengthy structural process management (in the hospital's higher-level system network)
	**Opportunities** (external) Increased efficiency of the treatment processes for patients, potential cost reductions (travel/absence costs, earlier decisions and start of treatments) Higher satisfaction of the patients, GPs and specialists involved with their daily work and employers Networking with civil institutions, opening up the health organization for "digital health solutions “	**Threats** (external) Data protection Hacking attacks, lack of funding approval

### BCG Matrix

In the BCG Matrix (Table 2), OVCs should still be considered a question mark. On the positive side, increasing acceptance by patients and encouragement from GPs as gatekeepers were recorded in the course of the project. At the same time, the share of online video consultations in the total number of outpatient contacts in the observation period was highly similar to the literature [6]. Especially in the given structures, OVCs have the potential to become a star and to positively influence the quality of treatment by streamlining existing processes and quickly bridging larger distances.

**Table 2:** BCG Matrix with the relative market share (X-Axis), potential market growth (in %, Y-Axis) and positioning of the Project “Online Video Consultation” as a “question mark”

**High market growth**	Question mark X	Star
**Low market growth**	Poor Dog	Cash Cow
	**Relative market share: low**	**Relative market share: high**

### Change management for OVC implementation

Various measures were taken to attract doctors and patients to the project. In addition to increased direct communication and communication with contacts, GPs were given the opportunity to book appointments online themselves. In case of questions or for short-term bookings, a new telephone number was set up for GPs and clinic doctors at the hospital, with a nursing team member who was available to them as a contact person. For ward physicians, separate handouts were created with instructions on how to book an OVC via the consult function in the hospital information system and brief instructions on how to use the OVC online program were offered. Finally, special handouts were prepared for patients to inform them about the possibility of an OVC upon admission to the hospital and instructions on how to contact the hospital if they were interested in an OVC for follow-up after discharge.

A detailed change management analysis according to Kotter [28] with 8 focal points for the project is presented in Table 3.

**Table 3:** Change management analysis of the implementation of OVCs according to Kotter [28]

1. Create a sense of urgency	This can be done by analyzing the respective actual situation of the clinic and extrapolated data of a possible OVC application (time savings, patient satisfaction and acquisition, etc.). From the experience gained, this is best done by convincing patients of the effectiveness and quality of OVCs from the ongoing process (e.g., interest of patients).
2. Establish a leadership coalition	The basis is the support of the clinical and commercial management of the clinic. Then, all specialists and senior physicians should be brought on board for their respective functional areas. In the course of the project, regular exchange and low-threshold support for the implementation must be ensured.
3. Develop a vision and strategy	In this project, the vision was to digitally support existing patient treatment. This was to be communicated to the staff on a regular basis. In perspective, there should be a strategy to expand the offer to other clinics, hospitals and health regions. Here, other digital tools should also be included to improve the treatment of patients and facilitate the work of staff.
4. Communicate vision of change	This takes place in personal discussions and further training, especially at the clinic but also in telephone calls and e-mails to the participating GPs.
5. Enable employees on a broader base	Doctors, as well as receptionists and nurses, are instructed in the use of OVCs to learn and adopt the technique.
6. Secure quick wins	For this purpose, planned OVCs can be used by a few enthusiastic specialists and selected patients with corresponding evaluations of their satisfaction and objective data acquisition (time saving, etc.).
7. Consolidate successes and initiate further change	Consolidation can be achieved by establishing and reinforcing the processes. Further applications, such as digital speech analysis or diagnostic support by means of artificial intelligence, will represent the future.
8. Fastening new approaches in the culture	A culture of digital support for the daily routine of doctors and nurses will become an important topic in the coming years in the respective clinic and beyond.

### Process management analyzes of the overall project

An analysis of the OVC project based on the 7 questions in process management according to Stöger [29] included the factors of result orientation, customer orientation, contribution to the whole, controllability/measurability/assessability, repeatability/routine, responsibility, manageability and is shown in Supplement 4.

**Fig. 3:**
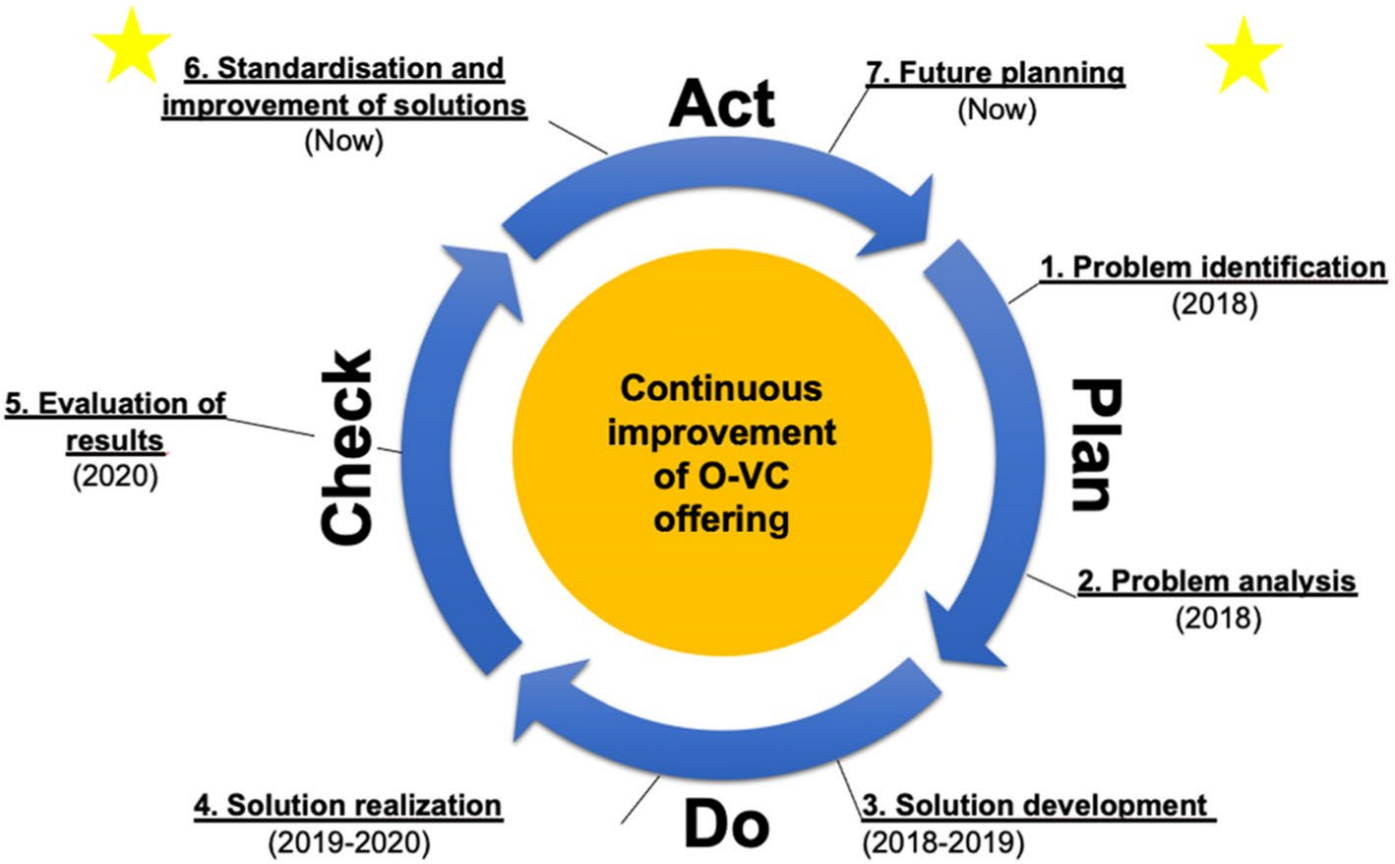
The PDCA cycle according to Deming, applied to the online video consultation project (Star-Mark: current status)

The following is an analysis of the project carried out using Deming's PDCA cycle in the total quality management approach (Fig. 3).

#### Planning (Plan)

- 1. Identification of problems
- Problems such as the loss of time in treatment processes, indirect and delayed communication between the clinic and GPs and sometimes long journeys (with consecutive absences from work, possibly the provision of an additional driver, etc.) were already identified in the run-up of the pilot introduction of OVCs.
- 2. Analysis of the problems
- Subsequently, it was examined to what extent the existing potential for improvement could be more easily improved by other measures. However, since the existing distances in particular could not be changed, it was additionally analyzed in the context of the existing literature and the assessment of experts on whether OVCs could also represent a relevant alternative in the field of orthopedics/trauma surgery.

#### Implementation (Do)

- 3. Development of solutions
- After OVCs were confirmed as showing potential, indications for the digital presentation of patients in the outpatient clinic were defined, and the project was prepared with partners in several steps. This was done in accordance with the legal framework, existing providers and technical implementation possibilities in the health network.
- 4. Realization of solutions
- The project was finally implemented in several steps within the framework of a pilot research project. Intensive feedback on technical improvements and adaptations on the part of the civilian providers, as well as discussions with patients and other medical users in the project, were ongoing from the beginning.

#### Testing (Check)

- 5. Evaluation of the results
- The collected results showed positive outcomes with a high acceptance rate among the participating specialists, GPs and patients. Other positive aspects of OVCs were revealed, such as closer networking and personal relationships among the medical players of the health region. At the same time, it was found that the resulting procedures from OVCs and "live" examinations hardly differed in the overwhelming majority of observed cases to objectively strengthen the benefits.

#### Action (Act)

- 6. Standardization of the solutions and continuous improvement
- Currently, the project is in the phase of standardizing the evaluated processes. At the same time, the first improvements are already being made in the sense of adapting the processes to achieve the best possible customer and staff orientation to achieve a high level of acceptance and thus consistency and sustainability of offering OVCs.
- 7. Planning for the future
- In addition to the core focus on the continuation of the existing service offer in the health region, parallel plans are already being drafted to expand the offer within the specialist disciplines in the hospital and expand to other regional GP practices. There are always close agreements among the responsible key players in organizational management.

## Discussion

Digitalization poses novel challenges to health care systems worldwide by supporting – in some cases, sometimes radically – changing existing mindsets, processes and structures [31, 32].

In this case, OVCs were introduced as a supporting link between orthopedic specialists, GPs and patients in a defined health region in eastern Germany to improve existing processes in the clinic/rural region interfaces in patient care.

In the existing approach, an overall positive conclusion could be drawn. The patients and doctors involved were, for the most part, very satisfied with the use of OVCs, in accordance with existing international data [33]. At the same time, the percentage frequency of use of OVCs, in relation to the total population of military patients treated in the outpatient clinic, was even slightly above the data of other international study data on OVCs at 9% [6]. The process analysis of the roll-out experience revealed several benefits and challenges, as discussed below.

As an implication for the regional establishment and expansion of offering OVCs in a (supra)regional health region, it seems sensible to map this within the framework of a process organization to enable spatial and temporal structuring of the processes as a basis for an organizational structure. To do so, responsibilities and strategic goals for OVCs need to be clearly defined and controlled in advance.

In the described case, Alfred D. Chandler's credo "*structure follows strategy*" was not fully followed [34]. While the goals and identification of resources were initially set, it was not possible to fully align the structure of the clinic and patient pathways in OVCs within the framework of this pilot project. It must therefore be discussed whether "*strategy follows structure*" does not apply, since the OVC project grew out of the recognized and addressed potential for the improvement of existing processes in the Bundeswehr Medical Service.

Total quality management was used as an important component for process optimization. Its application has often been successfully used to improve processes in the health sector [35] and represents an important framework structure in the presented project.

In the project, however, fixed setup structures were not initially established according to a traditional organizational approach [36], as the extent of use and implementation was not initially foreseeable. When planning an expansion of OVCs to other locations, a systematic definition of the processes of an OVC should take place for easier implementation [37].

In addition, a concrete process cost analysis [10] with a comparison of not only the costs (caused by travel/waiting time, personnel expenditure and technology for the implementation of OVCs) but also the revenue from online appointments and, in the medium and long term, with regard to the quality of treatment. If applicable, patient acquisition should be taken into account.

The involvement of staff in the sense of change management concepts, e.g., according to Kotter [28], should take place from the very beginning when introducing digital innovations in health care companies. According to the experience gained here, the implementing facilities can do without a permanent additional expenditure of personnel with the corresponding additional costs. Existing outpatient clinic staff can manage the organizational operation of online video consultations well based on the experience gained, especially if an online appointment booking system is linked to the respective hospital information system [38]. For a period of approximately 2 months, however, personal contact and supervision by a competent "facilitator" who is familiar with the system should be possible (e.g., from the provider company or from the system environment) to be able to immediately address procedural questions or problems and thus promote acceptance and willingness to use the system. In addition, staff should be provided with training and handouts, as well as email and telephone advice and support.

Using the example of regional cooperation between GPs and clinic outpatient departments ("local" hospitals), a new form of digital telemedical integrated care structures in a health region can be envisaged for the future. The described project can be considered a population-based model for a regional network [39]. Here, the exchange and cooperation between outpatient and inpatient care providers, general practitioners and specialists is digitally enabled. In this concept, military doctors act as gatekeepers for the Bundeswehr, which is especially important in rural areas with only a few regional hospitals compared to urban areas. In addition to networking through OVCs, digital communication channels such as email traffic and digital interfaces for the rapid transmission of images and findings between practices and hospitals must be created in parallel.

In health care companies with clinical and practice components, the use of OVCs and other digital services could further strengthen the idea of a company network as a cooperative patient-oriented overall system and bring the individual elements closer together [40]. This form of creating proximity through direct collegial consultative exchange was also positively emphasized by medical users within the framework of the current project. In this way, specialized medical providers could benefit the patients entrusted to them in the best possible way. At the same time, current professional standards could be safely used on a broad scale [41].

Looking at strengths and weaknesses of the current project, among the main strengths can be seen the evidence of avoidance of travel efforts and the acceleration of decision processes within patient care. Also, the improved interaction of medical key players like GPs and clinical specialists has to be enlightened as potential basis for a better networking and patient care. The SWOT and portfolio analysis confirmed the potential for improving existing health care processes by the use of OVCs. With an adequate change management strategy, process management approaches based on e.g. the PDCA cycle could be used for implementation of new digital telemedical approaches also in health care networks in the field of orthopedics.

However, in the course of the implementation of the OVC, also weaknesses of the chosen approach became evident. There was a learning phase, in which the GPs had to be won and trained for the use of the OVC. Repetitive consultative discussions took place between the project team and the GPs to explain the benefits and to motivate an ongoing use. The project experiences also showed, that for many months of implementation phase, a continuous support in organizational and technological structures has to be provided. Technical problems, such as insufficient mobile internet or communication-device malfunction, deserve an immediate problem management to ensure users´ compliance and adherence to new processes.

Finally, the study itself also had some limitations. First, the number of selected regional facilities and included patient cases was not large enough to make a differentiated statement for all potential existing orthopedic pathologies. In addition, the voluntary nature of participation in the study may have led to biased selection. Among doctors and patients, those with an interest in digital topics may have been more willing to participate in the project, resulting in a higher acceptance of this digital technique. In the future, more patients with different ages and a more balanced gender distribution should be included.

For the future, the first experiences of the here presented telemedical health network approach should be further followed up. Digital solutions have a high potential to support existing communication structures between patients and health care providers, but also in between the latter themselves. Decisive seems to be a scientific analysis and both multicenter and international exchanges about useful approaches for a digitally enhanced improvement of healthcare network based patient care.

## Conclusion

The introduction of online video consultations in a clinic with an associated care region is associated with a change in existing processes and should be addressed with strategic management approaches for the establishment of digital integrated care structures in a health region. It makes sense to apply the tools of total quality management and strategic control. Changes in management approaches are important to involve the staff in a timely manner and to support acceptance of the new measures.

## Supplementary Information

**Additional file 1. Supplement 1:** Comparative illustration of the established "old" processes and innovations using online video consultation in the follow-up of patients after inpatient treatment (placement marked in red).

**Additional file 2. Supplement 2:** Comparative illustration of the established "old" processes and innovations using online video consultation for the initial presentation of patients to specialists by general practitioners (placement marked in red).

**Additional file 3. Supplement 3:** Comparative illustration of the established "old" processes and innovations using online video consultation for the re-presentation of patients to specialists by general practitioners (placement marked in red).

**Additional file 4. Supplement 4:** Analysis of the existing online video consultation project according to the 7 factors with the central questions in process management formulated by Stöger.

## Data Availability

The datasets used and/or analyzed during the current here described research project are available from the corresponding author upon reasonable request.
