# Supplementary figures and images for: Implementation of online video consultations in a regional health network: a management feasibility analysis from an orthopedic perspective
Source: BMC Health Serv Res. 2022 Aug 12;22:1029. doi: 10.1186/s12913-022-08352-0 (PMC9372946; doi:10.1186/s12913-022-08352-0)

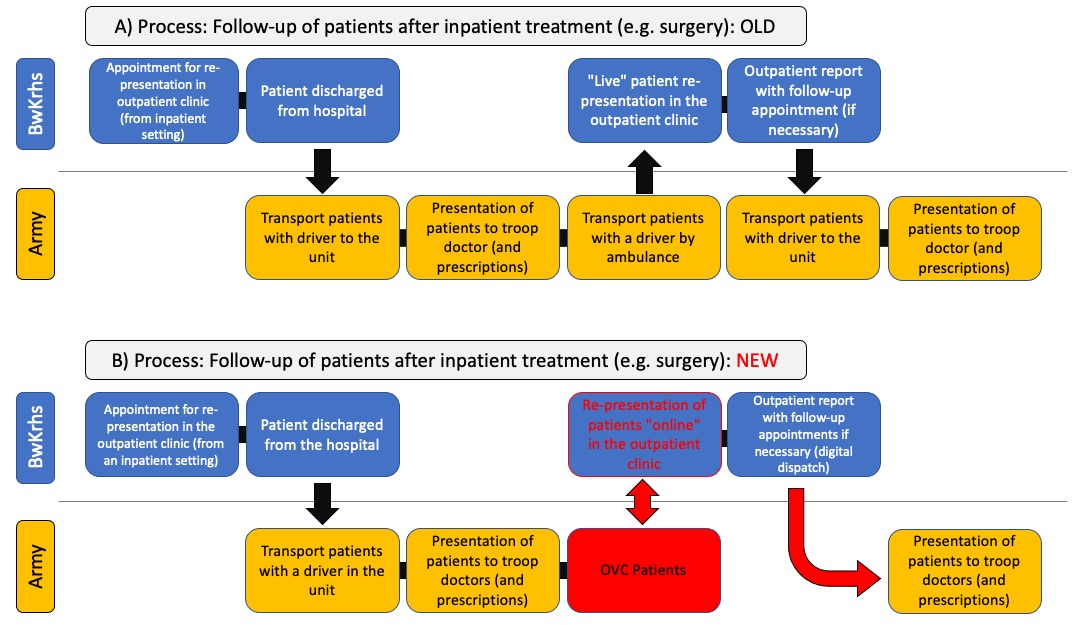

Supplement: Supplementary file 1 — Additional file 1. Supplement 1: Comparative illustration of the established "old" processes and innovations using online video consultation in the follow-up of patients after inpatient treatment (placement marked in red). [file 12913_2022_8352_MOESM1_ESM.jpg]

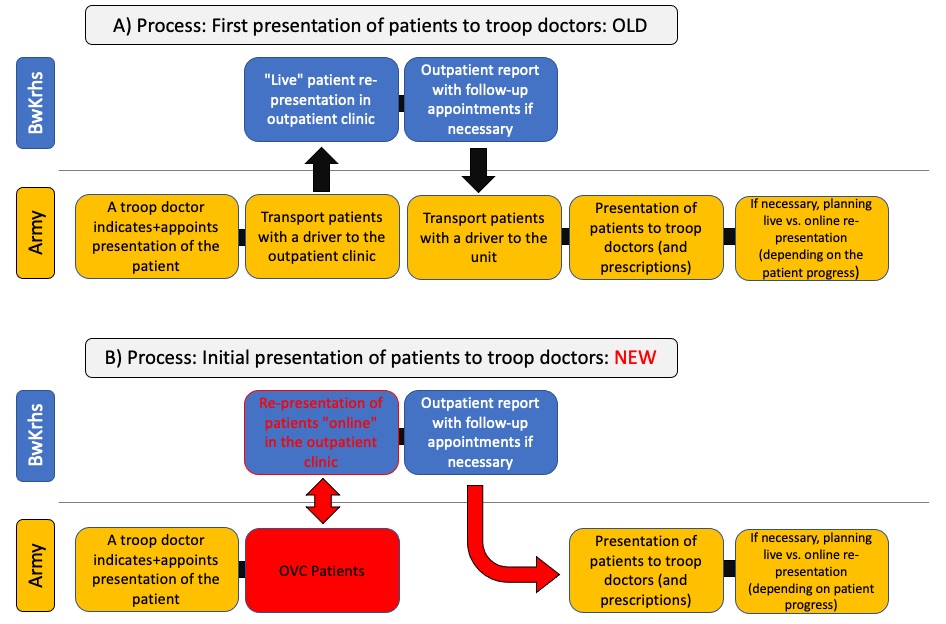

Supplement: Supplementary file 2 — Additional file 2. Supplement 2: Comparative illustration of the established "old" processes and innovations using online video consultation for the initial presentation of patients to specialists by general practitioners (placement marked in red). [file 12913_2022_8352_MOESM2_ESM.jpg]

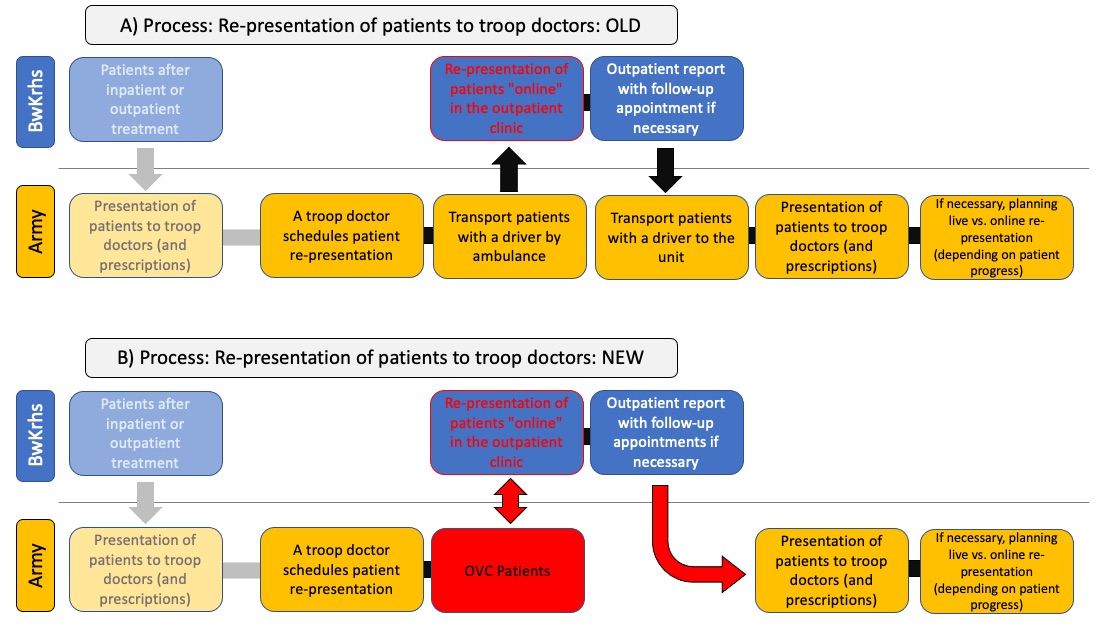

Supplement: Supplementary file 3 — Additional file 3. Supplement 3: Comparative illustration of the established "old" processes and innovations using online video consultation for the re-presentation of patients to specialists by general practitioners (placement marked in red). [file 12913_2022_8352_MOESM3_ESM.jpg]
