# Supplementary material for: Implementation of online video consultations in a regional health network: a management feasibility analysis from an orthopedic perspective
Source: BMC Health Serv Res. 2022 Aug 12;22:1029. doi: 10.1186/s12913-022-08352-0 (PMC9372946; doi:10.1186/s12913-022-08352-0)
Supplement: Supplementary file 4 — Additional file 4. Supplement 4: Analysis of the existing online video consultation project according to the 7 factors with the central questions in process management formulated by Stöger. [file 12913_2022_8352_MOESM4_ESM.docx]

Supplement 4

| **Result orientation** |
| --- |
| *Are there measurable and controllable results for each process?* |
| As part of the pilot study, evaluation data was collected for the advised processes, which is now already available as results. |
| *Are there clear objectives?* |
| For O-VS applications, objectives are clearly defined with indication-based digital replacement of in-person outpatient presentations. |
| *Are the definition of the objectives comprehensible and built into control systems?* |
| The definition of the objectives are comprehensible and control systems are specified by medical ethics and legal frameworks. |
| *Can specific deliverables be designated for each process?* |
| Yes, the service is always ultimately the preparation of a physician's report after a telemedicine consultation has taken place. |
| **Customer focus** |
| *Is the process evaluated by customers?* |
| While the pilot project provided for a constant evaluation of the participating patients and also troop physicians, this was no longer planned for the continuation of the project. The reason for this was that the effort involved in the evaluation survey was perceived as disruptive in some cases. In the future, evaluations among patients will be planned according to their needs and as part of regular feedback rounds with the GPs. |
| *Is there a definable customer group?* |
| Yes, the customer group can be clearly delimited by the affiliation of the patients with the German Armed Forces and the narrowing down to specific issues.. |
| *Do competitors and competitive processes exist?* |
| Due to the structure of the Bundeswehr, there are hardly any competitors here. The process itself is without competition in its current form, but is an integral part of the processes. |
| *Does the process logic also correspond to the customer's thinking?* |
| Yes, the logic of the processes corresponds to what the patients are used to or how it is given to them. The digital version of these processes is new, but due to the voluntary nature of its use, it is not considered disruptive. |
| **Contribution to the whole** |
| *Can upstream and downstream processes be clearly identified?* |
| Due to the processes already implemented and established in everyday clinical practice (patient presentation, diagnostics, conservative vs. surgical procedures, follow-up care), all the processes involved can be clearly identified. |
| *Can the contribution of the individual processes to an overall result be determined?* |
| Due to clearly delimitable processes with their own documentation (outpatient letter, discharge letter, rehab report, etc.), the contributions can also be clearly identified. |
| *Are there opportunities for rationalization by combining processes with sub-processes?* |
| In perspective, individual steps in which the personal presentation of patients would not be absolutely necessary can be saved. However, care must always be taken to ensure that this is justifiable from a specialist's point of view and must not be to the patient's disadvantage. |
| *Does process orientation prevent a fragmentation of forces or promote concentration?* |
| Yes, the project can bundle and concentrate forces. The concentration on fixed O-VC days in the outpatient clinic will also serve this purpose. |
| **Controllability, measurability, assessability** |
| *Is there systematic feedback on controllability, measurability, assessability?* |
| While in the pilot study feedback on the processes of O-VC was provided by both physicians and patients, in the future only a fixed evaluation of each case by the performing specialist of the outpatient clinic is planned (in order to maintain quality control on the one hand, and on the other hand not to impair the processes by obligatory evaluation of all parties involved in each case. |
| *Are tasks, competencies and responsibilities clearly defined?* |
| In the pilot phase, these points were clearly regulated within the study team. While they still exist now, it is foreseeable that further training and recruitment of nursing and medical staff should take place in order to secure the continuity of task fulfillment (especially appointment allocation and planning, implementation of O-VC). |
| *Is it possible to allocate costs, people and services to a process without overlap?* |
| Yes, due to the individual documentation of each case, an overlap-free assignment of each O-VC use is clearly possible. Theoretically, the respective working time of the physicians involved can also be clearly calculated accordingly. |
| **Repeatability, routine** |
| *With what does the process begin, with what does it end?* |
| The process of an O-VC begins with the scheduling of the O-VC by the attending GP or approaching the respective ward physician by an inpatient (or scheduling at the suggestion of the attending specialist) and ends with the completion of the documentation of an O-VC and its transmission to a GPs providing further treatment. |
| *How high is the standardization at the processing and control level of a process?* |
| While the content of each O-VC is very individual, the control level of scheduling, link sending, dial-in of patient/physician can be standardized very well (but depends on technical devices and internet connection). |
| *How high is task diversity and its representation in the processes?* |
| In this process currently under consideration, the variety of tasks is small, as they are clearly defined. However, follow-up decisions (e.g., unplanned further examinations, etc.) can expand the range of tasks for patients and physicians. |
| *How durable is a process once "adjusted"?* |
| The indication for O-VC is determined individually depending on the patient case. As a process itself, O-VC is now permanently implemented if indicated (with the standing option to change the process). |
| *How homogeneous is a process in itself?* |
| From a purely procedural point of view, with scheduling - sending links - online dial-in physician/patient - online contact - end of O-VC - documentation completion, every O-VC is homogeneous. |
| **Responsibility** |
| *Is the responsibility per process and sub-process in one hand?* |
| Ultimately not: The O-VC scheduling is determined by the unit physician or surgeon/ physician at hospital. It is desirable, but not always feasible, that the O-VC is then also performed by the same physician. |
| *How do information and communication work?* |
| It is important that the information about an O-VC scheduling takes place separately from a doctor's letter in a separate documentation (e.g. in the electronic hospital information system, KIS). In the future, patients should be informed of the fixed date and time of the O-VC upon discharge and an entry should be made in the KIS at the same time. Similarly, a reservation should also take place when an appointment is booked by unit physicians. The missing interface between the booking portal and the HIS should be addressed in the medium term. |
| *Are people interchangeable in principle?* |
| In principle, yes, with retention of known physicians being a relevant variable for patients both on the basis of literature sources and from their own experience to date in the pilot project. |
| *How high is the dependency on individual persons (regarding knowledge, experience)?* |
| Physicians should have gone through the process approximately 3 times before they would perform O-VC independently (not for technical reasons, but to anticipate or be able to address technical issues, for example, in the case of poor quality of dial-up) |
| *Can responsibilities be transferred to existing organizational units?* |
| This is at least the perspective goal, which is that O-VC use should be able to occur in other hospitals or other disciplines within the healthcare enterprise. |
| **Manageability** |
| *How pronounced is the implementation orientation in the individual processes and in the network?* |
| The implementation orientation currently still depends heavily on individual persons and their insistence on scheduling an O-VC (including the implementation). The use and booking of O-VC by unitphysicians cannot be influenced. In the future, the process should be completely independent of individuals, at least on the part of the clinic/outpatient department. |
| *Are both the processing level and the control level mastered?* |
| The processing level has already been tested. The control level is now being established as a follow-up to the pilot project, with fixed office hours for online video consultation. |
| *Can a process also be planned and controlled individually?* |
| The planning of individual O-VS can be planned and controlled individually. |
